# Understanding pediatric palliative care within interdisciplinary palliative programs: a qualitative study

**DOI:** 10.1186/s12904-023-01194-5

**Published:** 2023-06-24

**Authors:** Patricia Rico-Mena, Javier Güeita-Rodríguez, Ricardo Martino-Alba, Lourdes Chocarro-Gonzalez, Ismael Sanz-Esteban, Domingo Palacios-Ceña

**Affiliations:** 1 Department of Physiotherapy, Chiropody and Dance, Physical Therapy and Health Sciences Research Group, Universidad Europea de Madrid, C. Tajo, S/N, 28670 Villaviciosa de Odón, Madrid, Spain; 2 International Doctorate School, Rey Juan Carlos University, Madrid, Spain; 3 Department of Physiotherapy, Occupational Therapy, Rehabilitation, and Physical Medicine, Humanities and Qualitative Research in Health Science Research Group, Universidad Rey Juan Carlos, Madrid, Spain; 4 Pediatric Palliative Care Unit, Hospital Infantil Universitario Niño Jesús, Madrid, Spain

**Keywords:** Pediatrics, Palliative care, Patient care team, Child, Parents, Qualitative research

## Abstract

**Purpose:**

To describe the process of delivery of pediatric palliative care from the perspective of a pediatric interdisciplinary team and the children’s parents.

**Methods:**

A qualitative descriptive case study was conducted. Purposeful sampling took place within a specialized pediatric palliative care Unit in Madrid (Spain), located at the Niño Jesus Hospital. The study participants included a specialized pediatric palliative care team from Madrid's pediatric palliative care program, other professional teams involved in interdisciplinary care and parents of children under pediatric palliative care. Data were collected via semi-structured interviews, focus groups and researchers’ field notes. A thematic analysis was performed.

**Results:**

This study included 28 participants (20 women, 8 men), of whom 18 were professionals who belonged to the pediatric palliative care interdisciplinary team, 4 professionals were from other units that collaborated with the pediatric palliative care, and 6 were parents (5 women, 1 man). The mean age of the pediatric palliative care members was 38.2 years (SD ± 7.9), that of the collaborating professionals was 40.5 (SD ± 6.8), and that of the parents was 44.2 (SD ± 5.4). Two main themes emerged: a) Pediatric palliative care has a distinct identity, associated with *life*. It represents the provision of *special care* in highly complex children, in the context of the home, far from the hospital environment; b) The team is key: its interdisciplinary organization provides a more comprehensive view of the child and their family, fosters communication among professionals, and improves coordination with other services involved in the care of children. The mindset shift experienced by ID-PPC professionals towards a palliative approach makes them more sensitive to the needs of their patients and leads them to develop specific skills in areas such as communication, decision-making, and adaptability that were identified as differentiating aspects of pediatric palliative care.

**Conclusions:**

Describing pediatric palliative care from the professional and parental perspective helps to establish realistic and comprehensive goals for the care of children and their parents. The findings of this study may help with the establishment of a pediatric palliative care team, as a necessary organizational change in a health care system that cares for children with complex and life-threatening conditions. Promoting training in pediatric palliative care, prioritizing more horizontal organizations, providing tools and spaces for coordination and communication between professionals from different services, together with the creation of a position of case coordinator in the care process of children could enhance the understanding of pediatric palliative care services.

**Supplementary Information:**

The online version contains supplementary material available at 10.1186/s12904-023-01194-5.

## What is known

- A greater understanding of pediatric palliative care (PPC) facilitates greater availability and establishment of services; however, the role of PPC is not always clear among health professionals and parents.

## What is new

- Healthcare professionals and parents understand PPC thanks to the distinctive role that they assign to it, with a focus on quality of life and special care for children with complex and life-threatening conditions. The professional skills and attitudes of the interdisciplinary team help improve the understanding of these services.
- Better communication, the involvement of designated care coordinators and the provision of specific training courses, enhances understanding and favors greater cohesiveness of PPC.

## Introduction

Pediatric palliative care (PPC) applies to children and adolescents with life-limiting/life-threatening conditions (LL/LTC) [1, 2]. In the last decade, PPC has experienced notable improvements [3], however, there is still a lack of specialized services [4–6]. Thus, in clinical practice, many children who could benefit from PPC do not receive this service, or care is delayed [4, 7–13]. In clinical practice, PPC continues to be mistakenly identified with end-of-life care, hindering its delivery [11, 14, 15].

In PPC, six levels of development are described [6]. Spain is at the third level, characterized by isolated, irregular, and insufficient provision. In the European Atlas of PC [4], Spain appears in the third quartile, ranking at the 31st place out of the 51 countries analyzed, with 0.6 specialized PC services compared to the 2 points of the countries in the first quartile [4]. Furthermore, knowledge and dissemination of PPC facilitates its development [1, 7, 16]. In addition, the perception of health professionals and families on PPC facilitates and influences its implementation [7, 14, 15, 17]. What essential elements of PPC are shared by members of an interdisciplinary PPC team (ID-PPC) and the parents of the children receiving such care? The aim of this study was to describe the functioning of PPC from the perspective of a pediatric interdisciplinary team and parents of children receiving PPC.

## Methods

This study was conducted according to the Consolidated Criteria for Reporting Qualitative Research [18] and the Standards for Reporting Qualitative Research [19].

### Design

A qualitative case study was undertaken [20, 21]. This design comprises different participants, contexts, places and moments, connected by the phenomenon under study [20, 22]. In this study, the phenomenon is the process of care for children included in the Madrid PPC program, as experienced by the different participants (including parents and professionals). In Madrid, PPC is currently provided through the only specialized PPC Unit that exists in this community, which is located at the Niño Jesus Hospital (NJH).

### Participants, sampling strategies, and sample

Purposive sampling and snow-ball techniques were conducted (November 2017 to June 2020), aimed at including those participants who possessed information that was relevant to the study [23]. Sixteen participants were recruited by purposive sampling (November 2017 to July 2019) and 12 were recruited by snowball sampling (September 2019 to June 2020). Participants were accessed based on the institutional email of all professionals who were members of the ID-PPC and professionals collaborating with the ID-PPC. Thus, a formal email was sent to prospective participants describing the study and requesting their participation. In the case of parents, contact was made through the social workers of the ID-PPC from the specialized unit of NJH. Telephone sessions were conducted with the parents to explain the nature of the study and to request their participation. Participant recruitment and data collection ceased when the information gained from the interviews became repetitive [24]. The authors agreed that the information would be considered repetitive when there were sufficient in-depth data showing themes, categories, and patterns of the studied phenomenon. To accomplish this, the authors reviewed the analysis and the quality of the participant quotes that were obtained [24]; in our study this situation occurred after including 26 participants. Subsequently, two additional participants were included to confirm that data saturation had indeed been reached [24].

This study included professionals working within the ID-PPC, professionals from other teams that collaborate in the application of PPC, and parents of children receiving PPC. The inclusion criteria consisted of: a) being a member of the ID-PPC (at the time of the study); b) other professionals collaborating with the ID-PPC, being health professionals from other units of the NJH, who participate in PPC; c) parents, having or having had children included in the NJH PPC program. All participants were requested to sign the informed consent form. A total of 21 ID-PPC members and 30 professionals collaborating with the ID-PPC were invited to participate via email. Among the ID-PPC members, 20 professionals responded to the email and expressed interest in participating. Among the other professionals collaborating with the ID-PPC, only four responded to the email and participated. The reasons for not participating are unknown. As for the parents, 10 parents participated in the meetings, however, only six parents showed interest in participating.

### Data collection

In-depth interviews, focus groups and researchers' field notes were used [25] (Table 1).

**Table 1:** Data collection instruments

	**Participants**	**Data collection tool**	**Time (minutes)**
Professionals of the team specialized in PPC	PCD, PCN, PCP, PCSW, PCPT, PCA	18 Semi-structured interviews and field notes	Mean: 58 min
Other professionals that collaborate in the application of PPC	Neuropediatricians and rehabilitators	1 focus group	Mean: 50 min
Parents	Fathers and mothers	1 focus group and 1 semi-structured interview	Focus Group; Mean: 50 min Interview: 120 min

*PCD* Specialist Palliative Care Doctor, *PCN* Specialist Palliative Care Nurse, *PCP* Specialist Palliative Care Psychologist, *PCSW* Specialist Palliative Care Social Worker, *PCPT* Specialist Palliative Care Physiotherapist, *PCA* Specialist Palliative Care Administrative, *PPC* Pediatric palliative care

A series of questions was established for each group of participants based on the literature and expert knowledge (Tables in the Supplement, Tables S1, S2 and S3). A guide of semi-structured questions was applied for the interviews and focus groups. The researchers explored the meaning of PPC and the process of applying PPC for the profile of each participant. The interviews and focus groups were conducted in Spanish and were audio-recorded after obtaining permission from the participants.

### Data analysis

A transcription of each interview, focus group and researcher's notes was made. An inductive thematic analysis of the transcribed texts was conducted by three researchers (PRM, JGR, DPC). The coding and identification of themes was carried out through the reading of the overall text, as well as paragraph by paragraph, and an in-depth line by line analysis. The coding was carried out in distinct phases: 1) Generation of initial codes; 2) codes were grouped into categories [26, 27]. The categories were then grouped, and sub-themes and topics were identified. 3) Review of themes, re-coding and identification of new themes and construction of conceptual maps of the themes. 4) Identification and definition of themes and sub-themes, establishing their definition [28]. The Atlas-ti® (v.8) qualitative analysis software was used [29] and the Excel® program [30].

### Rigor and quality criteria

The criteria for guaranteeing trustworthiness by Guba & Lincoln were applied [31]. The techniques used to control trustworthiness are reported in Table 2. The use of these methods to increase rigor are compatible with case-study designs [32, 33].

**Table 2:** Trustworthiness criteria

Criteria	Techniques Performed and Application Procedures
Credibility	Investigator triangulation: Team meetings were organized during the thematic analysis, the results were compared, and the final results were identified Member checking: post interview patient member checking consisted in offering all participants the opportunity to review the audio or written records
Transferability	In-depth descriptions of the study, providing data and describing the study design and its different sections (context, research team, reflexivity process, sampling, inclusion criteria, data collection, and analysis)
Dependability	Audit by an external researcher, responsible for the assessment of the study protocol, with a special focus on the method and process of implementation during the study
Confirmability	Investigator triangulation, data collection triangulation. The process of reflexivity was conducted through the description of the researchers’ positioning; reflective debriefing by the researchers during data collection and analysis

### Ethical considerations

This study was approved by the Research Ethics Committee of the Rey Juan Carlos University ( code:2606201710917) and the Clinical Research Ethics Committee of the Niño Jesús Hospital (code: R-0019/17). Furthermore, this study was conducted in accordance with the principles of the Declaration of Helsinki. Informed consent and permission to record the interviews were obtained from all participants.

## Results

Twenty-eight participants (20 women, 8 men) took part in this study. Thus, the sample included 18 professionals who were part of the ID-PPC, four professionals from other units collaborating with the PPC, and six parents (5 women, 1 man). The mean age of the ID-PPC members was 38.2 (SD ± 7.9), that of the collaborating professionals was 40.5 (SD ± 6.8), and that of the parents was 44.2 (SD ± 5.4 years). See Tables in the Supplement, Tables S4, S5 and S6 for the sociodemographic data of the participants. It should be noted that there is an over-representation of ID-PPC members, which may influence the results by obtaining a predominantly “professional” perspective of the studied phenomenon, overshadowing the perspectives of other participant groups (see limitations section).

**Fig. 1:**
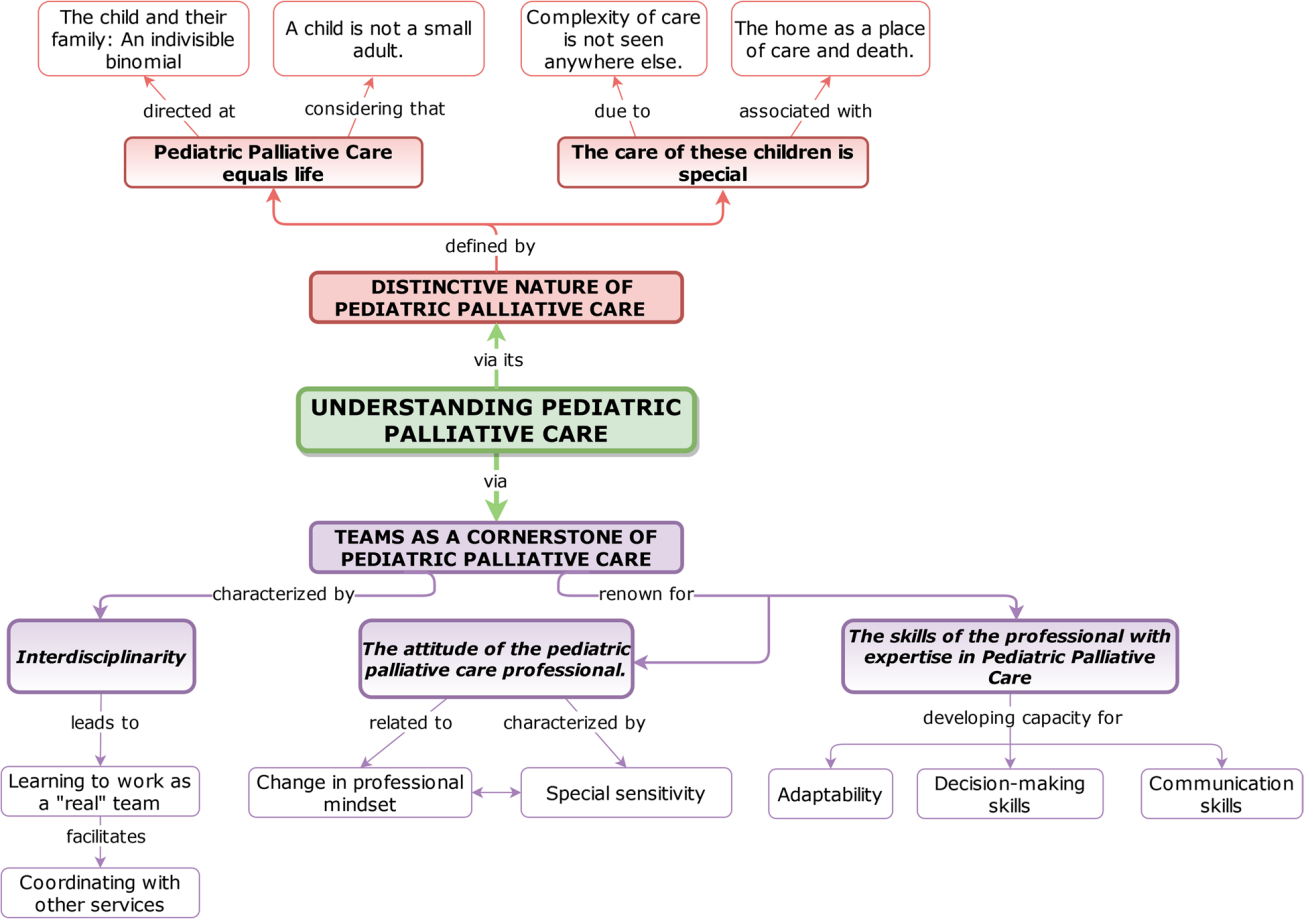
The hierarchies and relationships between the themes and sub-themes

Two main themes were identified, with five subthemes (Table 3). The hierarchies and relationships between the themes and subthemes appear in Fig. 1. In Table S7 examples of participants' narratives are shown to justify the results obtained, thereby enabling their traceability and credibility [34].

**Table 3:** Themes, subthemes, and categories

Themes	*Subthemes*	Categories
**Theme 1. Distinctive nature of Pediatric Palliative Care**	*Subtheme 1: Pediatric Palliative Care equals life*	Category 1: The child and their family: An indivisible binomial Category 2: A child is not a small adult
	*Subtheme 2: The care of these children is special*	Category 1: Complexity of care is not seen anywhere else Category 2: The home as a place of care and death
**Theme 2: The team as a cornerstone of Pediatric Palliative Care**	*Subtheme 1: Interdisciplinarity*	Category 1: Learning to work as a “real” team Category 2: Coordinating with other services
	*Subtheme 2: The attitude of the pediatric palliative care professional*	Category 1: Change in professional mindset Category 2: Special sensitivity
	*Subtheme 3: The skills of the professional with expertise in Pediatric Palliative Care*	Category 1: Adaptability Category 2: Decision-making skills Category 3: Communication skills

Our results show how the distinctive nature of PPC, together with the contributions of the ID-PPC team and other professionals, form the basis for understanding PPC and give meaning to these services.

### Theme 1. The distinctive nature of pediatric palliative care

ID-PPC practitioners associated PPC equaling *life* rather than death. The frailty and complexity of the children cared for means that they require *special care* within their home. Thus, participant PCN5 described the nature of pediatric palliative care:

*“Everyone thinks of palliative care and thinks of death, they think of drugs that are given when you die, they think of terminal sedation. But nobody thinks about the years of life, that they have a quality of life, nobody thinks about how a child with cerebral palsy eats, nobody thinks about the hours that parents spend at the hospital because their child is sick, nobody thinks about the interim, everybody thinks about death. I think that is the most invisible thing.”*

#### Subtheme: pediatric palliative care equals life

PPC entails comprehensive care for children and their families, which is different to PC for adults. PPC is provided to children with incurable diseases, emphasizing the quality of life throughout the course of the illness. It also focuses on ensuring a quality death and supporting the family's life after the loss of their child.

### The child and the family: an indivisible binomial

The professionals highlighted the need to include all the people in the child’s environment. As they are minors and dependent, the family is fundamental because parents need to be trained to care for the children. Professionals understand PPC as care while living, as opposed to the perception of PPC in relation to death. The death of the child is accepted as an inevitable outcome, however, PPC focuses on the child's day-to-day life, avoiding interventions that increase the child's suffering or unnecessary hospital stays. The accompaniment of the family during bereavement after the death of the child differentiates PPC from other disciplines that terminate their services at the death of the child.

*“The meeting with the palliative care team was like a soothing balm for me. It was not just about discussing the child, but about all of us. By 'talking,' I mean taking measures for the entire family, so that we can all be in a better place because if I'm not well, I can't take care of my daughter.” (M5).*

### A child is not a small adult

The professionals point out that there is a tendency to extrapolate adult PC criteria to children. Caring for children is more complex than caring for adults, as it includes a wide variety of symptoms and diagnoses, with the presence of non-oncologic pathologies, such as neurologic pathologies, which have a longer survival from the onset of PPC.

*“A child, being a child, seems to me to be a fragile person who needs the care of his or her family, so the health care of a child and an adult is different; it is not a small adult…” (PCSW2).*

#### Subtheme: the care of these children is special

Children receiving PPC are considered special, as they present diseases of great *symptomatic complexity,* requiring centralized care at the* home.*

### The complexity of care is not seen anywhere else

Complexity is defined by the use of technology, the management and adaptation of pharmacology (designed for adults) to the children’s profile. Experience and professional training are key to master this complexity, turning ID-PPC into experts in symptomatic control of discomfort and suffering. For professionals it is hard to deal with symptoms such as pain, dyspnea, or seizures.

*“They have very specific care, they have devices that I as a nurse did not know about until I arrived here, with a complexity of care that is not seen anywhere and also the other more spiritual part of accompaniment is not taught anywhere and it is essential.” (PCN4).*

### The home as a place of care and death

The home is the place of choice for parents for caring and death. This implies greater adaptation and effort on behalf of professionals. For parents, home care is “a luxury” and the child’s condition makes transportation a challenge. For professionals, working in the home makes it easier to understand the family dynamics, establish greater trust, and cover needs in a timely manner. On occasions, ID-PPC professionals perceive that some parents “misuse” this care, by changing the planned visitation schedule for unjustified reasons.

*“It's different being in the hospital with a barrier table, everything feels colder, compared to when you're sitting on a couch in a home with the mother beside you, sharing life stories and problems. But when you have such a close relationship, they often call us to schedule a visit and ask to change the time or day because they have something else. It's okay, but I can assure you they don't call the oncologist and say, 'Hey, I won't come at ten because I have a physiotherapy appointment, I'll come at one.' It's a bit two-faced.” (PCD5).*

### Theme 2. The team as a cornerstone of pediatric palliative care

The ID-PPC is the cornerstone of care, characterized by interdisciplinarity, professional attitude and the development of specific skills.

#### Subtheme: interdisciplinarity

ID -PPC professionals emphasize that belonging to the PPC team means learning to work in a “real” team. Coordination and collaboration with other professionals is a cornerstone of PPCs.

### Learning to work as a “real” team

The participants concurred that within ID-PPC there is greater coordination compared to other medical services. The professionals emphasized that PPC is based on a group approach to the child, not an individual one. PPC is based on creating meeting spaces that help shared decision making, empathy, and respect. Leadership plays a key role in ID-PPC, and all participants agree on the urgent need to incorporate professionals who are not present in healthcare settings in Spain, such as psychologists and social workers, because they enhance the overall understanding of each child and consequently improve care.

*“What surprised me the most when I came here was the way they work as a team here. For me, the reason that this works is because we work as a “real” team. The fact that there is a psychologist, a social worker, a doctor, a nurse, that they know the child and they all have a say. This is the only place where, for the first time, I have heard a doctor say to the nurse: “What do you think about this?” or “What do you think we have here?” (PCN4).*

### Coordinating with other services

Parents become coordinators of the care received from the multiple medical specialties that their children need. They agree that ID-PPC improves the coordination of care, although it does not completely resolve it. They demand the need for a case coordinator for non-pharmacological therapies such as physiotherapy or speech therapy. Thus, the parents felt that in services such as rehabilitation, traumatology, neurology and pneumology, there is a lack of consensus among professionals and contradictory information is conveyed, causing them to feel “lost”.

*“I think that there is still an important part that should be coordinated, and that isn’t happening right now, the issue of physiotherapy treatments, etc., it seems that doctors do not want to get involved in it, it seems that physiotherapists are something else. Some have even said “that it doesn't do anything”, “that it’s just as good as drinking a glass of water”, […] Then there are many hours in the day and you need many recommendations that doctors don't give you, postural, exercises… And right now, this isn’t coordinated…” (F1).*

#### Subtheme: the attitude of the professional with expertise in pediatric palliative care

ID-PPC professionals emphasized the need for a change of mentality to enable professionals to be flexible, proactive, decisive, and willing to work as a team. This change of mentality is thanks to the continuous contact with children and their families, which helps the professional to develop greater sensitivity of the reality of the child and the family.

### Change in professional mindset

The ID-PPC professionals described how their attitude towards the child's illness and death changed, accepting death as a natural event and not as a professional failure. They focused their efforts on providing comfort to the child and the family and not on providing a cure. This change emerges when they must endure the suffering of children when PPC is not applied. All professionals agreed that this change must occur for their own emotional well-being.

*“What sets palliative care apart is that it acknowledges that the child is going to die, whereas other professionals may not accept this. By accepting that the child is going to die and having prior knowledge of it, you can change the way you care for them based on their needs. The approach to care includes how you inform them, the expectations you create, adapting to the reality, prioritizing the quality of daily life, and ensuring that the child's best interests guide decision-making, rather than focusing solely on the diagnosis or available treatments.” (PCD4).*

### Special sensitivity

Parents considered that the professionals working in PPC were “different”. They used adjectives such as “special”, “human”, “sensitive” when caring for children who suffer. The professionals pointed out that it is necessary to have a special sensitivity for PPC, although the majority agreed that an adequate and specialized training allows any professional to provide good PPC. PPC should not be associated with an individual’s good qualities but rather with having a good qualification in PPC.

*“I think the human quality of all the people who are in palliative care is greater, that' s what I've seen, the work they do, is that of a special person, who has a special vision of life because he knows death very closely. I think all the members of the palliative team had that quality. I don't know why, because they aren't trained… or maybe they are trained, I don't know, but I certainly miss the fact that all the doctors have that attitude and empathy.” (M5).*

#### Subtheme: the skills of the professional with expertise in pediatric palliative care

PPC involves the development of new skills to provide an adequate response to children’s needs. Among others; adaptive capacity, decision making in extreme situations, communication (bad news), and pharmacology management.

### Adaptability

The ability to adapt makes it possible to respond to complex and changing situations in PPC such as home care, which removes the professional from the security of the hospital environment. Home care is an unpredictable environment, with fewer resources, where the professional adapts to the “rules of each home”. Resourcefulness and flexibility allow the professional to adapt to different social and cultural realities.

*“…because when we go to a hospital it is the patient who adapts to the rules of the healthcare environment. When we go home, we are the ones who must make an effort to adapt to hundreds of different families: a doctor who goes—a team that goes—on the same day to four families, with each family you have to adapt to their way of being, of being, their home, their internal family rules, their style of communication …” (PCD4).*

### Decision-making skills

Decision-making in PPC is a shared responsibility between professionals and parents. In ID-PPC, it falls on the physician. Decision-making is facilitated in the presence of specific training and experience in PPC, active listening to individual needs and the correct establishment of priorities and goals. Parents reported that participating in decision making generates anxiety and insecurity, and they need continuous advice from professionals and coordination of the information they are given.

“I feel more accompanied and that they provide me with better explanations to make decisions (since we are in palliative care), although you realize that medicine isn't an exact science, so sometimes there is no right and wrong decision….” (M2).

### Communication skills

Events of failed communication is a source of conflict between the team and the parents. For professionals, having good communication skills reduces the emotional impact of bad news on children and their parents, and strengthens the professional-patient bond. Parents highlighted how PPC professionals have better communication skills due to their training and experience.

*“When I talk about different communication and communicative abilities, you are dealing with patients and families who are extremely fragile. Any communication error can potentially cause a rupture in the physician–patient relationship that may be difficult to repair due to time constraints and the emotional impact it creates. To achieve a balance between direct and honest communication, while being empathetic and respectful, it requires a broad knowledge of communication techniques and the ability to quickly recognize the communicative needs of each individual.” (PCD6).*

The professionals acknowledged the need to incorporate spaces for communication to facilitate meetings among professionals. Examples of this include periodic team meetings, and the child death and family bereavement meetings. Parents were surprised that all professionals from the ID-PPC knew about their child's situation. The professionals pointed out that the car was a widely used space for informal communication*.* During the commute to work, the car is a place where conflicts are addressed, and bonds are strengthened among the professionals on the team.

## Discussion

Our results reveal the perspectives of professionals and parents on PPC. This type of service is focusing on life, the interdisciplinary nature of the team, collaboration, and the development of specific skills, which are considered key elements of PPC.

Our results highlight how the views of professionals and parents are complementary in many aspects. Parents made comments that are relevant to the functioning of an ID-PPC, such a good communication, good training, greater sensitivity and flexibility. Previous studies [11, 35] describe how there is a misinterpretation of PPC, interpreting it as end-of-life care for the child [11], or such care is only associated with the relief of physical symptoms [35]. PPC means care during life and not only upon the child's death [7]. Our participants pointed out the importance of an early application of PPC, and the use of specific scales in children to help and justify referral at earlier stages in relation to the adults [36].

Our results show how parents recognize ID-PPC professionals as key players in the care of children. Specialized PPC services are necessary for the coordination of care and symptom control between the hospital and home teams [37]. Parents of children with cancer experience great difficulties in coordinating appointments and maintaining contact with the professionals themselves due to the lack of a designated person to coordinate care with the PPC team [38].

The parents in our study recognize ID-PPC professionals as key personnel in their children's care. They describe how the lack of communication between professionals and services is one of the biggest problems they face, being perceived as a threat to their children's health [39, 40]. Parents of children with cancer experience great difficulties in coordinating appointments and maintaining contact with the professionals themselves due to the lack of a designated person to coordinate care with the PPC team [38]. The uncoordinated care described by parents has been previously recognized in the literature [38, 39, 41, 42], reflecting an unresolved problem in healthcare systems. Specialized PPC services are needed for coordination of care and symptom control between hospital and home teams [37]. We hope that our findings will help to rethink the way in which care is provided to this group, as they describe the need for a staff representative to coordinate care within a health system that is fragmented into medical specialties, promoting communication and collaboration among the various health care providers. The construction of spaces for communication and coexistence is another noteworthy finding. It is to be expected that this information promotes informal coexistence spaces, such as the car or home care, since they favor communication.

Parents of children with LL/LTC conditions need time for an in-depth conversation based on trust avoiding prejudice and value judgments [43]. Insufficient communication skills in professionals can become barriers to quality PPC [44–47]. The success of PPC depends on the seamless sharing of information among team members [48], and improves symptom control in children with cancer [49]. In addition, Meert et al. [50] found that bereaved parents valued their physicians' communication skills when there was use of plain language, clarity and pacing of information, consistency of information, honesty, and empathy demonstrated in verbal, nonverbal, and affective communication.

In light of our findings, we suggest the need for professionals to foster a humane, empathetic, humble, respectful and flexible attitude in the care of parents and children in PPC. Our results show that the attitude of professionals towards PPC is fundamental in the care of parents and children. Walter et al. [47] identified how professional attitudes, together with training and management of the uncertainties, helped professionals to transition from a curative to a palliative mentality. Previous studies [17, 47, 51–53] show how professional attitudes, sensitivity and experience directly influence clinical practice, patients’ feelings and parents’ decision making. In addition, Bergstraesser et al. [37] show that flexibility of the professionals to adapt the treatment to the place and the needs of the child and the family is a characteristic of the PPC.

This study has certain constraints on the generalizability of findings, which limit the extrapolation of our results. One of the limitations of this study is the over-representation of the group of professionals belonging to the PPC team. The authors believe that this could influence the data obtained and influence the results by obtaining a “professional” perspective of the phenomenon under study, over other perspectives of other groups such as “other professionals” and “families”. The authors were unable to include more participants from the other groups, because in the case of the “other professionals” group, the number of professionals collaborating with the PPC team was very limited. In the case of the family group, they had many difficulties because they declined to participate in the study, due to personal reasons. In addition, the authors controlled the possible influence of the “PPC professional” group on the results through researcher triangulation during analysis and data collection, and by using external auditing (see Table 2). Also, it should be noted that in the qualitative case study, it is also key to include participants with different profiles in order to enrich the results and obtain further information and understanding of the phenomenon under study [54, 55].

Another limitation is the collection of information from different data collection instruments applied to the groups of participants. Thus, in the group of PPC professionals, in-depth interviews were applied, while in the groups of other professionals and the family, focus groups were used. The use of different data collection instruments could influence the results obtained. Moreover, because qualitative case studies are a design that integrates multiple perspectives on a phenomenon under study, the use of different data collection instruments is encouraged to obtain as much information as possible from all possible qualitative data sources [54, 56]. In addition, the authors found it very difficult to collect data individually (time constraints in the research schedule) among the group of other professionals and family members. For this reason, another data collection instrument was applied, focus groups, which made it possible to collect relevant information on the phenomenon under study, optimizing time, from participants who met the inclusion criteria and who had key information on the phenomenon under study [25].

Nonetheless, our results can help health professionals to better understand PPC and the perspectives of professionals and patients regarding the PPC process, and the relationship between PCC´s professionals and families.

## Conclusions

Understanding the meaning and characteristics of PPC from the perspectives of parents under PPC and professionals working in this area generates greater understanding of the PC process in children.

Our findings have the potential to enhance communication within interprofessional relationships by highlighting the importance of adopting less hierarchical organizational structures and providing training in both PPC and effective communication skills. These factors can serve as a foundation for redesigning programs aimed at children in PPC.

The establishment of coordinated collaboration between specialized PPC teams and other medical services or departments is crucial. Furthermore, the inclusion of a dedicated case manager within these teams is essential to facilitate improved communication between parents and healthcare professionals. Future research endeavors should concentrate on assessing the communication skills of professionals, identifying factors that influence the effectiveness of ID-PPC, and exploring avenues for bolstering family support.

## Supplementary Information

**Additional file 1: Table S1.** Semi-structured question guide for professional members of the interdisciplinary pediatric palliative care team. **Table S2.** Semi-structured question guide for professionals collaborating in the implementation of Pediatric Palliative Care. **Table S3.** Semi-structured question guide for parents.** Table S4.** Sociodemographic data of participants from the interdisciplinary PPC team.** Table S5.** Sociodemographic data of participants who were professionals collaborating with the interdisciplinary team.** Table S6.** Sociodemographic data of parents participating in this study. **Table S7.** Narratives from themes and subthemes.

## Data Availability

The datasets generated and/or analyzed during the current study are not publicly available due to ethics restrictions but are available from the corresponding author on reasonable request.

## Abbreviations

ID-PPC: Interdisciplinary PPC team
LL/LTC: Life-limiting/life-threatening conditions
NJH: Niño Jesus Hospital
PC: Palliative care
PPC: Pediatric palliative care
