# Supplementary material for: Understanding pediatric palliative care within interdisciplinary palliative programs: a qualitative study
Source: BMC Palliat Care. 2023 Jun 24;22:80. doi: 10.1186/s12904-023-01194-5 (PMC10290414; doi:10.1186/s12904-023-01194-5)
Supplement: Supplementary file 1 — Additional file 1: Table S1. Semi-structured question guide for professional members of the interdisciplinary pediatric palliative care team. Table S2. Semi-structured question guide for professionals collaborating in the implementation of Pediatric Palliative Care. Table S3. Semi-structured question guide for parents. Table S4. Sociodemographic data of participants from the interdisciplinary PPC team. Table S5. Sociodemographic data of participants who were professionals collaborating with the interdisciplinary team. Table S6. Sociodemographic data of parents participating in this study. Table S7. Narratives from themes and subthemes. [file 12904_2023_1194_MOESM1_ESM.doc]

### Understanding pediatric palliative care within interdisciplinary palliative programs: A qualitative study.

Patricia RICO-MENA^1,*^ MsC, Javier GÜEITA-RODRÍGUEZ^2^ PhD, Ricardo MARTINO-ALBA^3^ PhD, Lourdes CHOCARRO-GONZALEZ^3^ PhD, Ismael SANZ-ESTEBAN^4^ PhD, Domingo PALACIOS-CEÑA^2^ PhD.

^1^ Universidad Europea de Madrid. Faculty of Sport Sciences. Physical Therapy and Health Sciences Research Group. Department of Physiotherapy, Chiropody and Dance. Spain; International Doctorate School, Rey Juan Carlos University, Madrid, Spain; ([patricia.rico@universidadeuropea.es](mailto:patricia.rico@universidadeuropea.es))

^2^ Humanities and Qualitative Research in Health Science Research Group. Department of Physiotherapy, Occupational Therapy, Rehabilitation, and Physical Medicine, Universidad Rey Juan Carlos, Spain ([javier.gueita@urjc.es](mailto:javier.gueita@urjc.es); domingo.palacios@urjc.es)

^3^ Pediatric Palliative Care Unit, Hospital Infantil Universitario Niño Jesús, Madrid, Spain. ([ricardojavier.martino@salud.madrid.org](mailto:ricardojavier.martino@salud.madrid.org); lourdes.chocarro@oberon.es)

^4^ Universidad Europea de Madrid. Faculty of Sport Sciences. Physical Therapy and Health Sciences Research Group. Department of Physiotherapy, Chiropody and Dance. Spain. ([ismael.sanz@universidadeuropea.es](mailto:ismael.sanz@universidadeuropea.es))

*Corresponding author: Patricia RICO-MENA

Universidad Europea de Madrid. (Department of Physiotherapy, Chiropody and Dance. Physical Therapy and Health Sciences Research Group), C. Tajo, s/n, 28670 Villaviciosa de Odón, Madrid, Spain

Email: [patricia.rico@universidadeuropea.es](mailto:patricia.rico@universidadeuropea.es)

Table S1. Semi-structured question guide for professional members of the interdisciplinary pediatric palliative care team.

Table S2. Semi-structured question guide for professionals collaborating in the implementation of Pediatric Palliative Care.

Table S3. Semi-structured question guide for parents.

Table S4. Sociodemographic data of participants from the interdisciplinary PPC team

Table S5. Sociodemographic data of participants who were professionals collaborating with the interdisciplinary team.

Table S6. Sociodemographic data of parents participating in this study.

Table S7. Narratives from themes and subthemes.

Table S1. Semi-structured question guide for professional members of the interdisciplinary pediatric palliative care team.

| **Research areas** | **Questions** |
| --- | --- |
| **The meaning of PPC** | What does PPC mean to you? and to other healthcare professionals?  What differentiates PPC from other specialties?  What aspects of PPC do you consider to be the most unknown? |
| **The referral process** | What determines whether a child is included in a PPC program?  Where do you think it is easier or more difficult to get a referral from?  In your opinion, what factors facilitate or hinder the referral of a child to PPC?  What do you think is needed for PPC to be further developed? |
| **Care for children with palliative needs and their families** | How do you experience having to care for children whose prognosis is death?  How do families react when their child is proposed for referral to PPC? |
| **Self-perception of the professional role in PPC** | How do other professionals rate your work at PPC?  What do you think is the perception that families have of your work within PPC? |
| **Communication and decision making** | How is information handled and shared within the team? What is most relevant?  How is the process of communication with the family? How is the decision-making process carried out? |

PPC: Pediatric Palliative Care.

Table S2. Semi-structured question guide for professionals collaborating in the implementation of Pediatric Palliative Care.

| **Research areas** | **Questions** |
| --- | --- |
| **The meaning of Pediatric Palliative Care** | What does pediatric palliative care mean to you?  Could you describe to me in what parts of your daily clinical practice do you apply elements of PPC (skills, content, etc.)?  Which elements of PPC do you find the most relevant and the most difficult to apply?  To what extent do you believe you meet the palliative needs of the pediatric patients you care for?  How might receiving specific training in PPC influence your clinical practice?  How is the relationship and coordination with the interdisciplinary PPC team established?  What do you consider that specialized PPC resources contribute to your work? |
| **The referral process** | What criteria must appear in order to consider a child you care for to be referred to PPC?  Could you describe critical situations (events, incidences) that you have had in the process of referring a child to PPC?  Do you think there are adequate standardized criteria to guide the referral of children to PPC?  Do you believe that all the children you serve who are eligible for PPC eventually receive it? What can make it difficult or easy to access a specialized PPC program? |
| **Care for children with palliative needs and their families** | What experience have you had in caring for children with palliative needs? |
| **The process of communication and decision making** | How is the communication with the PPC interdisciplinary team? What is most relevant? How is the decision-making process carried out? |

PPC: Pediatric Palliative Care.

Table S3. Semi-structured question guide for parents.

| **Research areas** | **Questions** |
| --- | --- |
| **The care process of a child with palliative needs** | What is your experience with the care received from the PPC interdisciplinary team? |
| **The referral process** | How did you feel when you were first told that your child should or could be cared for at PPC? |
| **Meaning of Pediatric Palliative Care** | Did you know about the existence of PPCs before the referral? What did PPCs mean before you received this care?  What do PPCs mean now, and do you consider that your vision of PPCs has changed? What has been the most relevant aspect of PPCs for you? |
| **The process of communication and decision making** | How is the communication with the professionals of the PPC interdisciplinary team?  Do you participate in the decisions that are made regarding the treatment/management of your children? |

PPC: Pediatric Palliative Care.

Table S4. Sociodemographic data of participants from the interdisciplinary PPC team

| **Participant code** | **Profession** | **Sex** | **Age** | **Level of specific PC training** | **Time working at the PPC unit of NJH** | **Area or service where you previously worked** |
| --- | --- | --- | --- | --- | --- | --- |
| PCD1 | Pediatrician | Man | 45 | Masters | 2 years | Pediatric intensive care and emergency care  Pediatric liver transplantation  Pneumology  Neonatology |
| PCD2 | Pediatrician | Man | 33 | Masters | 3 years | Neuropediatrics |
| PCD3 | Pediatrician | Man | 35 | Masters | 5 years | Pediatric Intensive Care |
| PCD4 | Pediatrician | Man | 54 | Masters and PhD | 10 years and 7 months | Pediatric intensive care, general pediatrics, pediatric trauma, pediatric oncology, pediatric oncology |
| PCD5 | Pediatrician | Woman | 35 | Masters | 5 years | Neuropediatrics |
| PCD6 | Pediatrician | Woman | 32 | Masters | 4 years | Pediatric emergency medicine and general pediatrics |
| PCD7 | Pediatrician | Woman | 35 | Masters | 6 years | Has always worked at PPC |
| PCN1 | Nurse | Woman | 46 | Masters | 6 years | Catalan Institute of palliative oncology for adults |
| PCN2 | Nurse | Woman | 31 | Postgraduate course | 7 years | Pediatric oncohematology service |
| PCN3 | Nurse | Woman | 49 | Postgraduate course | 11 years | Pediatric emergency care, neonatology, intensive care and pediatric oncology |
| PCN4 | Nurse | Woman | 29 | Residency | 15 months | Pediatric intensive care, general pediatrics, pediatric trauma, pediatric oncology, pediatric oncology |
| PCN5 | Nurse | Man | 26 | Residency | 3 months | Pediatric nursing |
| PCP1 | Psychologist | Woman | 48 | Masters in family therapy/Basic course in PC | 5 years | Oncohematology and pediatric hematopoietic transplant service and pediatric intensive care. |
| PCP2 | Psychologist | Woman | 40 | Masters | 10 years | Hematology and hemotherapy |
| PCSW1 | Social worker | Man | 29 | Postgraduate course | 4 years | Pediatric palliative care foundations and adult palliative care service |
| PCSW2 | Social worker | Woman | 41 | Postgraduate course | 10 years | Pediatric Oncology |
| PCPT1 | Physical therapist | Woman | 37 | Postgraduate course | 6 years | Respiratory physical therapy for healthy children and children with associated pathology |
| PCA1 | Administrative staff | Woman | 42 | Basic course | 11 years | Pathological anatomy, neurology and social pediatrics |

PCD: Specialist Palliative Care Doctor; PCN: Specialist Palliative Care Nurse; PCP: Specialist Palliative Care Psychologist; PCSW: Specialist Palliative Care Social Worker; PCPT: Specialist Palliative Care Physiotherapist; PCA: Specialist Palliative Care Administrative; PC: Palliative care; NJH: Niño Jesús Hospital.

Table S5. Sociodemographic data of participants who were professionals collaborating with the interdisciplinary team.

| **Participant code** | **Profession** | **Sex** | **Age** | **Level of specific PC training** | **Time working at NJH** |
| --- | --- | --- | --- | --- | --- |
| PM&R1 | Physiatrist | Woman | 56 | PC course in adults | 23 years |
| PM&R2 | Physiatrist | Woman | 37 | No | 4 months |
| PM&R3 | Physiatrist | Man | 40 | No | 3 years |
| NP1 | Neuropediatrician | Woman | 29 | No | 1 year |

PM&R: Physiatrist; NP: Neuropediatrician; PC: Palliative care; NJH: Niño Jesús Hospital.

Table S6. Sociodemographic data of parents participating in this study.

| **Participant code** | **Relationship with the child** | **Age** | **Diagnosis of the child** | **Sex of child** | **Child's age (years)** | **Time spent in palliative care (months)** |
| --- | --- | --- | --- | --- | --- | --- |
| F1 | Father | 50 | | Hypoxic-ischemic encephalopathy and West Sd |  | | --- | --- | |  | | Girl | 14 | 10 |
| M1 | Mother | 49 |  |  |  |
| M2 | Mother | 37 | Cerebral palsy, spastic quadriparesis, refractory epilepsy/West syndrome | Girl | 3 | 12 |
| M3 | Mother | 44 | Polymalformative syndrome. Neurological involvement | Boy | 8 | 8 |
| M4 | Mother | 40 | Acute lymphocytic leukemia | Boy | 6 | 6 |
| M5 | Mother | 45 | Grade IV glioblastoma | Girl | 6 | 2 |

F: Father; M: Mother

Table S7. Narratives from themes and subthemes.

| **Theme 1. Distinctive nature of pediatric palliative care.** |
| --- |
| ***Subtheme: Pediatric palliative care equals life.*** |
| **The child and the family: an indivisible binomial** |
| *PCD3: “In any palliative unit, whether for adults or children, the unit is the patient and the family. However, in the case of children, this binomial is even more powerful. And, in addition, it is not only the parents, but also the siblings, it is the concept of the family. In palliative care you treat the child and the family; in other words, the rest of the medical specialties forget about the family, in fact, I believe that families are perceived by the rest of the doctors as - maybe it' s very pretentious to say, and this is my perception of the rest of the doctors - they see the family as a nuisance.”*  *PCP1: "Here, grief support is part of the care process. When a child passes away, we continue to be concerned about how the family is doing. We make a visit after three weeks, and there are follow-up calls. The team wants the psychologists to inform them about the family's well-being, if they have found employment, how they are adjusting... In other services, when a child passes away, it's the end. Here, we reach out to them, visit the funeral home, attach a condolence letter with the report, hold a session to analyze the care process and examine how it has emotionally affected us. We take care of the professionals in that aspect because we know we are working with children who die and their families, and there is a personal toll from the suffering we experience as well."* |
| **A child is not a small adult** |
| *PCN1: “The number of illnesses treated is much greater and these illnesses are treated for a longer period of time than what generally occurs in adults. Caring for a child means caring for an age range that goes from a newborn to a young adult. Therefore, there is a psychomotor, cognitive and emotional development, which makes it much more complex.”*  *PCD2: "I believe the type of patients we care for in pediatric palliative care is often unknown. We have patients who do not have cancer, unlike adult palliative care. I don't think people imagine children in wheelchairs with feeding tubes when they think of palliative care."* |
| ***Subtheme: The care of these children is special.*** |
| **Complexity of care is not seen anywhere else** |
| *PCPT1: “They are very complicated children. One of the difficulties that I have encountered the most is everything that a child with such a serious pathology, in terms of feeding through the feeding tube, all the devices they have, all the complexity of their condition... these children have so many things wrong, things that we can't control at all, medication, nutrition, many things that are beyond our control, the complexity of the disease in itself.”*  *PM&R3: "For example, I am incapable of managing the medication for these children. The management of complex medication that palliative care professionals handle is something I cannot handle myself because I am not a pediatric palliative care physician."* |
| **The home as a place of care and death** |
| *M2: “She also has home care, which is very convenient for us, because we transport her with oxygen 24 hours a day, she is aspirated 50 times a day, so we go with oxygen with a portable aspirator, we have to aspirate her, take out the tube, clean it so that it doesn't get dirty. To move around with her is a huge effort, the chair weighs 50 kg, it weighs as much as I do. So, for me, to have them come to my house is a real treat.”*  *PCD4: “I think that sometimes families do not take the doctor very seriously, because they don't wear a lab coat and they are at their home, whereas they bend over backwards when the doctor wears a tie and is at the hospital...But the effort to play the role of a doctor, a nurse, a psychologist or a social worker in a home is much greater than at hospital [...] the effort to adapt, but at the same time making it clear that we are the healthcare team responsible for your child, is a very big effort; it requires an effort of flexibility and having some flexibility to change, to distance oneself, to become closer, to adapt to a family with a high economic status or in exclusion, on the same day the same team has to adapt to so many different families.”* |
| Theme 2. The team as a cornerstone of pediatric palliative care. |
| ***Subtheme: Interdisciplinarity.*** |
| **Learning to work as a “real” team** |
| *PCD1:"Before working here, I had never experienced such a strong sense of teamwork. Even when I worked in an ICU and was the only one on duty with a nurse, the work was much more compartmentalized. I had never worked so closely with a social worker and a psychologist. To me, it seems incredibly important that everyone, including physiotherapy, or even the administrative staff, whom I consider part of the team because they are so essential..."*  *PCP1: “I believe that the boss acts as a common thread or some sort of glue, he makes things work better, but because of the way he directs them, because I have had other experiences where the work was more chaotic. I think the boss has made us all take into account the work of others and what is possible, and that we work as a team, he' s like the conductor of the orchestra, each musician plays, and the conductor of the orchestra makes it harmonious ...”.* |
| **Coordinating with other services** |
| *M2: “I have missed a figure that I don't think exists, which is someone who coordinates. You are suddenly faced, in our case, with seven different specialists and you have to make decisions that are very important for your child's life and about which you have no idea, you don't know what is best, you don't have an understanding of what is best, you don't have knowledge… “.*  *PM&R2: "The palliative care team helps us coordinate, they assist us in coordinating... For example, I am currently seeing many children who have been without rehabilitation follow-up from our side for two years, and palliative care has redirected them back to us."* |
| ***Subtheme: The attitude of the professional with expertise in PPC*** |
| **Change in professional mindset** |
| *PCD3: "It's about changing one' s mind-set. I am a very pragmatic person, I believe that health professionals suffer because they don't change their mindset. If you continue thinking as a doctor who cures, this is hell because everyone dies, but if you change your objective and your objective is not that someone lives, that is, not that they live longer, but that they live better and you achieve it, well, of course, they die, and the family suffers, and part of that suffering gets to you, but once you understand that it' s something normal, that it will happen to all of us and that we are all going to go through it, in the end you say: "okay, they're going to suffer, but if I intervene they're going to suffer less than if I don't intervene," and so it becomes something positive."*  *PCN3: “…I saw children dying in very unpleasant circumstances [...] That's when I said: "This isn't normal, we can't ignore the fact that we aren't really doing things right. That child was very introverted, he didn't talk to anyone, he didn't say anything to his parents because he knew he was dying, he was bedridden, so you say: "My God, I can' t believe that we aren't capable of helping people to die, at the end of their days". And that's when something clicked and I began to investigate, to learn about palliative care…”* |
| **Special sensitivity** |
| *M3: "They are different, they approach illness in a different way, starting from the perspective of: 'What do we care for?' We care for quality of life. They may not explicitly say it, but we don't prolong your child's life; we try to make their days better, free of pain. So, I believe they are different professionals because they are part of a team that is different from all the others."*  *PCN5: “Of course there is a sensitivity and I believe that this sensitivity is what makes us get involved in this, but I probably wouldn't have this sensitivity if I hadn't received prior training. The basis is that there should be good training, and that' s going to differentiate the quality of the care we provide. People are very different, but we all have a certain sensitivity, probably because we have undergone this experience, because we have been trained in it, because we have realized that this need exists. So, it' s not that we are special here and that we are guardian angels.”* |
| **Subtheme: The skills of the professional with expertise in PPC** |
| **Adaptability** |
| *PCP2: “…you have to be very flexible, not be dependent on a context, on the practice, on your place of safety. Here the consultation is either the hospital room or your home. So, you have to know how to establish a good link from the beginning with the child and his family members so that you can have a successful psychotherapeutic intervention. I believe that there are professionals who don't have this ability to adapt and therefore it' s a handicap for them and they can't step out of the clinical environment.”*  *M5: “When they came home, the meetings were led by different professionals depending on the day. For example, the nurse would lead the meeting on one day, the doctor on another day, and on the day when the topic of how the child would die was discussed, the psychologist would take the lead. This flexibility, where the necessary professional takes the main role in each situation, is unimaginable in regular medical services. They alternated the spotlight, and I believe it was adapted to our specific needs on that particular day. If it was more logistical, involving equipment, then the social worker would take charge. If it was more emotional and we were in tears, the psychologist would step in. If we were overwhelmed by corticosteroids, then the doctor and nurse would be there to help.”* |
| **Decision-making skills** |
| *PCD7: “It' s by experience, because you know the diseases better and you know how they are going to evolve, so if you know what's going to happen in the following months you can foresee whether or not they will benefit from comprehensive care at home, or if for the time being they won't benefit from anything new at home that they wouldn't be getting at the doctor's office.”*  *PCD3: "The physician should play a leading role in the diagnostic and therapeutic process of the patient, assessing their condition and deciding on the treatment to be administered. They should also serve as the primary communicator with the family, especially when delivering bad news, and take on the primary responsibility of making difficult decisions."* |
| **Communication skills** |
| *PCD4: “In fact, one family left our care because they perceived that whoever was taking care of them wanted the child to die quickly, and this is a child who is still living now, so they left palliative care. It was very early on, but let's say that what was conveyed to the family -although it wasn't the intention- wasn't perceived as such by the family, quite the opposite. So, there was a miscommunication, relationship issues, conveying messages....”*  *PCD6:” … I think the key moment is in the car. Everything can happen there, from you throwing a tantrum when you leave a house, to laughing like there's no tomorrow, or simply conveying your worries about patients that you see that day or that you saw the day before and didn't have time to discuss [...] The car gimmick is useful for everything: it helps me to let off steam, it helps me to disconnect, and above all it helps me with things that you haven't seen, in other words, the fact that in the same situation there are two people visualizing the scene makes your vision much broader.”*  *M1: “We had a very bad experience with the way they gave us the news of what was wrong with the baby at birth. My husband was only told that the baby had fetal distress without explaining anything else and at the hospital the neurologist told him this with very little tact […]. In my case, they had no empathy since it was the first day, I met my daughter after everything we had been through. Regarding palliative care, it is completely different, they explain things as they are, and within that reality, in a different way. For us, the palliative care team tells us how our daughter is, for better and for worse, and when things are very bad, they always do so in a close and empathetic manner […] In the last episode of bronchospasm we were explained his situation, each step taken and the reason for each step. We were asked if we preferred to be at home or in the hospital, and we were given both options explaining the pros and cons. You feel the support as doctors, explaining all the treatment from the beginning and also before applying it, they explain the reasons and ask you questions, and as people, they give you a hug when you need it”.* |
|  |
